# Serum leucine-rich α-2-glycoprotein and monocyte chemoattractant protein-1 as novel biomarkers for predicting vedolizumab response in patients with ulcerative colitis

**DOI:** 10.1093/crocol/otag084

**Published:** 2026-08-11

**Authors:** Takeo Yoshihara, Shinichiro Shinzaki, Tetsuji Naka, Hideki Iijima, Yoshito Hayashi, OGF study group, Takahiro Inoue, Yoshiki Tsujii, Ryotaro Uema, Yuriko Otake-Kasamoto, Mizuki Tani, Takahiro Amano, Taku Tashiro, Akiko Asakura, Yuko Sakakibara, Takuya Yamada, Satoshi Hiyama, Tsutomu Nishida, Kaori Ishiguro, Yuji Baba, Tetsuo Takehara

**Affiliations:** Department of Gastroenterology and Hepatology, The University of Osaka, Graduate School of Medicine, Osaka 565-0871, Japan; Department of Gastroenterology, Hyogo Medical University School of Medicine, Hyogo 663-8501, Japan; Institute for Biomedical Sciences Molecular Pathophysiology, Iwate Medical University, Iwate 028-3694, Japan; Division of Allergy and Rheumatology, Department of Internal Medicine, School of Medicine Iwate Medical University, Iwate 028-3695, Japan; Department of Gastroenterology and Hepatology, The University of Osaka, Graduate School of Medicine, Osaka 565-0871, Japan; Osaka International Medical & Science Center, Osaka Keisatsu Hospital, Osaka 543-0042, Japan; Department of Gastroenterology and Hepatology, The University of Osaka, Graduate School of Medicine, Osaka 565-0871, Japan; Japan Medical Office, Takeda Pharmaceutical Company Limited, Tokyo 103-8668, Japan; Japan Medical Office, Takeda Pharmaceutical Company Limited, Tokyo 103-8668, Japan; Department of Gastroenterology and Hepatology, The University of Osaka, Graduate School of Medicine, Osaka 565-0871, Japan; Department of Gastroenterology, Kansai Rosai Hospital, Hyogo 660-8511, Japan

**Keywords:** colitis, ulcerative, inflammatory bowel disease, leucine-rich α-2-glycoprotein

## Abstract

**Background and Aims:**

Effective, minimally invasive biomarkers are needed to predict treatment outcomes in ulcerative colitis (UC). This multicenter observational study investigated predictive serum and genetic biomarkers for vedolizumab (VDZ) therapy in UC, including serum leucine-rich α-2-glycoprotein (LRG), cytokines, and differentially expressed genes (DEGs) in intestinal tissue.

**Methods:**

Eligible patients (18-80 years; moderately to severely active UC) received intravenous VDZ (300 mg; Weeks 0, 2, 6, then every 8 weeks through Week 54). Serum biomarkers were compared between those who did/did not achieve clinical remission (CR; full Mayo score ≤2, all subscores ≤1) or mucosal healing (MH; Mayo endoscopic subscore ≤1) at Week 14. Week 0 inflammatory tissue data were stratified by Week 14 VDZ effect (CR and complete-MH [Mayo endoscopic subscore = 0]) for DEGs.

**Results:**

Overall, 21 patients were included. Mean serum LRG was significantly lower in the CR (*n* = 11) versus non-CR (*n* = 10) group at Week 0 (19.0 vs 29.0 µg/mL; *P* = .0267) and Week 6 (13.4 vs 19.6 µg/mL; *P* = .0340), and in the MH (*n* = 14) versus non-MH (*n* = 7) group at Week 6 (13.7 vs 21.6 µg/mL; *P* = .0326). Mean monocyte chemoattractant protein-1 (MCP-1) was significantly different between CR and non-CR groups at Week 0 (609.0 vs 403.4 pg/mL; *P* = .0018) and MH versus non-MH at Week 0 (587.2 vs 358.9 pg/mL; *P* < .001) and Week 6 (606.9 vs 451.6 pg/mL; *P* = .037). No significant differences in C-reactive protein and fecal calprotectin levels were observed. DEGs included *LRG1*, LRG-related cytokines, and *MCP1*.

**Conclusions:**

Serum LRG and MCP-1 may be candidate biomarkers of VDZ treatment response in UC.

## Introduction

Ulcerative colitis (UC) is an inflammatory bowel disease (IBD) characterized by recurrent flare-ups and remissions. Vedolizumab (VDZ) is a recombinant humanized immunoglobulin G1 monoclonal antibody that specifically binds to human lymphocyte α4β7 integrin, a plasma membrane protein on lymphocytes.^1^ Mucosal addressin cell adhesion molecule-1 (MAdCAM-1) is a transmembrane glycoprotein that is strongly expressed on the vascular endothelial cells of Peyer’s patches, mesenteric lymph nodes, and high endothelial venules of the intestinal intrinsic layer.^2^ Binding of VDZ to α4β7 integrin inhibits the interaction between α4β7 and MAdCAM-1, thereby reducing lymphocyte trafficking to the intestinal mucosa and associated lymphoid tissues.^1^ The global phase 3 GEMINI 1 trial demonstrated that VDZ is effective in inducing and maintaining clinical remission (CR), as well as significant mucosal healing (MH), in patients with UC.^3^

The clinical response rate to VDZ therapy in real-world cohorts of patients with IBD is 40%-60% in the induction phase.^4^^,^^5^ Therefore, it is necessary to identify the patient characteristics that increase the likelihood of a positive response to this treatment. Identifying predictive biomarkers, ie, pretreatment indicators of disease course and/or treatment response, could enhance therapeutic selection for patients and increase long-term remission rates. Unfortunately, effective and clinically useful predictive biomarkers are currently scarce. Variability in therapeutic responses suggests that the effectiveness of VDZ may be influenced by distinct immunological pathways predominant in individual patients.^6^ Components of these pathways, including serum inflammatory cytokines and chemokines, present promising candidates for novel predictive biomarkers. Furthermore, serum leucine-rich α-2-glycoprotein (LRG) at Week 0 can predict the effectiveness of ustekinumab treatment at Week 8 in patients with IBD.^7^ Although serum LRG is approved and widely used as a biomarker to evaluate disease activity for UC and Crohn’s disease in Japan,^8–10^ it has not been adopted widely internationally, and there is a lack of data for the role of serum LRG as a predictive biomarker for VDZ treatment. It is also possible that predictive biomarkers may be identified by investigating differentially expressed genes (DEGs) in the intestinal tissue of patients with UC with and without a clinical response to VDZ.

The purpose of this study was to explore predictive serum and genetic biomarkers for VDZ treatment in patients with UC, using serum LRG and serum cytokine assays and tissue transcriptome analyses to identify DEGs.

## Materials and methods

### Study design

This multicenter, open-label, single-arm, observational study evaluated the relationship of potential biomarkers, including serum LRG, serum cytokines, and DEGs identified by tissue transcriptome profiles, with clinical outcomes in patients with moderately to severely active UC treated with intravenous VDZ (300 mg; Weeks 0, 2, 6, then every 8 to 54 weeks). The study period was February 2021 to February 2023. The primary end point was the association between serum LRG concentration and treatment response at Week 14. Secondary and exploratory end points included the association between serum LRG concentration and treatment response at Week 54, the association of longitudinal change in serum biomarker concentrations with clinical outcomes, and the association between gene expression levels in colon tissue and clinical outcomes. The study was registered with the UMIN Clinical Trials Registry (Identifier: UMIN000043185).

### Ethical considerations

This study was conducted in accordance with the International Council for Harmonisation E6 Guideline for Good Clinical Practice, the Declaration of Helsinki, and ethical guidelines for medical research involving human subjects. The study was approved by the Institutional Review Boards at each site, and all patients provided written informed consent prior to the initiation of study procedures.

### Study population

Eligible patients were aged 18-80 years, were diagnosed with UC ≥3 months prior to enrollment, had moderately to severely active disease (defined as a partial Mayo score ≥4 and rectal bleeding ≥1 within 28 days prior to the first dose of VDZ), and had previously received corticosteroids, immunomodulatory drugs (azathioprine or 6-mercaptopurine), or biologics (tumor necrosis factor [TNF]-α antagonist or ustekinumab), or had prior failure of treatment with tofacitinib.

Major exclusion criteria were treatment with ustekinumab or infliximab within 8 weeks, golimumab within 4 weeks, adalimumab within 2 weeks, Janus kinase inhibitors within 1 week before the first dose of VDZ, or prior treatment with VDZ, natalizumab, efalizumab, or anti-CD20 antibody (eg, rituximab). Patients were excluded if they had any of the following: UC confined to the rectum; history of extensive colon resection, subtotal or total resection; history of ileal anastomosis, colostomy, or known symptomatic bowel stricture; active infection within 1 month prior to first VDZ administration; history of current or past malignancy; and history of hypersensitivity or allergies to VDZ or VDZ excipients and other criteria that, in the opinion of the investigator, would prohibit study participation. Pregnant and lactating women were also excluded.

### Treatment response groups

CR was assessed at Weeks 14 and 54. CR was defined as a full Mayo score ≤2 and all subscores ≤1. Patients were stratified into CR versus non-CR groups at both time points (individuals with missing Mayo subscore(s) were classified as non-CR). MH was also assessed at Weeks 14 and 54, and was defined as a Mayo endoscopic subscore ≤1 (individuals with a missing Mayo endoscopic subscore were classified as non-MH). Patients were stratified into MH versus non-MH groups at both time points. An individual patient could belong to both the CR and MH groups.

The CR/non-CR and MH/non-MH groups were used in the analyses of partial Mayo score response over time and levels of potential biomarkers over time.

For tissue transcriptome analyses, patients were stratified by Week 14 CR (CR vs non-CR) and Week 14 complete-MH (complete-MH vs non-complete-MH). Complete-MH was defined as a Mayo endoscopic subscore of zero. Patients with missing Mayo subscore information were excluded from these analyses. An individual patient could belong to both the CR and complete-MH groups.

### Assays and biomarker measurements

Blood samples (3 mL) and stool samples were collected from patients before administration of VDZ at Weeks 0, 2, 6, 14, 30, and 54 and used for measurement of serum biomarkers and fecal calprotectin (FC) levels. Serum LRG and serum C-reactive protein (CRP) were measured using latex agglutination turbidimetric immunoassays, FC was measured with a fluorescent enzyme immunoassay, and serum MAdCAM-1 was measured with an enzyme-linked immunosorbent assay. The serum cytokines measured were monocyte chemoattractant protein (MCP)-1, interleukin (IL)-1β, IL-6, IL-12/IL-23 p40, IL-8, IL-17A, IL-10, interferon (IFN)-γ, TNF-α, oncostatin M (OSM), eotaxin, IL-1α, IL-2, IL-5, IL-13, IL-12 p70, IL-22, IL-17F, and IL-23 p19. Assays used were the MILLIPLEX® human cytokine/chemokine/growth factor panel A (a 48-plex premixed magnetic bead panel; Sigma-Aldrich, St Louis, MO) for MCP-1, IL-1β, IL-6, IL-12/IL-23 p40, IL-8, IL-10, IFN-γ, TNF-α, eotaxin, IL-1α, IL-2, IL-5, IL-13, and IL-12 p70; the MILLIPLEX® human Th17 panel (a 25-plex premixed panel; Sigma-Aldrich, St Louis, MO) for IL-17A, IL-22, IL-17F, and IL-23 p19; and the MILLIPLEX® human cardiovascular disease panel (a 12-plex configurable panel; Sigma-Aldrich, St Louis, MO) for OSM. A false discovery rate (FDR) correction was not applied to the cytokine panel data.

Serum VDZ concentrations were measured using electrochemiluminescence, from samples collected at Weeks 2, 6, 14, 30, and 54.

At each collection time point, serum biomarkers, FC levels, and serum VDZ concentration were compared between those who did and did not achieve CR (CR vs non-CR group at Weeks 14 and 54) and those who did and did not achieve MH (MH vs non-MH group at Weeks 14 and 54).

### Transcriptome analysis

Tissue biopsies were collected from inflammatory sites at Weeks 0 and 14. Tissue samples were stored in RNAlater (Thermo Fisher Scientific, Waltham, MA) and processed using the RNeasy Mini Kit (Qiagen, Hilden, Germany). RNA integrity was assessed using the Agilent RNA 6000 Nano Kit on a 2100 Bioanalyzer (Agilent Technologies, Santa Clara, CA). Ion AmpliSeq™ Transcriptome Human Gene Expression Kit was used to prepare AmpliSeq libraries with 10 ng of RNA, and the libraries were sequenced using Ion 550™ Chip Kit and Ion 550™ Kit-Chef, and GeneStudio™ S5 Prime System (Thermo Fisher Scientific, Waltham, MA).

Identification of DEGs was performed by stratifying Week 0 inflammatory tissue data based on Week 14 CR and Week 14 complete-MH. FDR correction was not applied to the genetic data. The count matrix of all samples was converted to counts per million and log transformed (base 2), and 0.1 was added to genes with mean values >0 for all samples (removal of low-expression genes). The data were then normalized using the trimmed mean of M values method. Finally, the R package limma 3.50.3, with its voom method, was used to identify DEGs. The thresholds for identification were a *P* value <.05 and a fold change >1.5.

The biological function of DEGs in relation to clinical outcomes in patients treated with VDZ was characterized using enrichment analysis. Enrichment analysis was performed using R package enrichR_3.1 and the Reactome_2022 library. Terms were identified using a *P* value <1 × 10^−5^ as the cutoff criterion. The negative log of the *P* value (–logP) was calculated.

To identify specific signaling pathways related to the VDZ treatment effect, network analysis of the DEGs was performed using STRING v11.5 (https://string-db.org) and Cytoscape 3.10.1 software.

### Statistical analysis

The mean differences and confidence intervals of the partial Mayo score, serum biomarkers (LRG, CRP), FC, serum MAdCAM-1, serum VDZ concentration, and serum cytokines/chemokines at each evaluation time point were calculated based on the treatment effect (CR and MH) at Weeks 14 and 54. The Welch *t*-test was performed to test the difference of the means. All missing values were classified as non-remission. For biomarkers with significant differences between CR and non-CR or MH and non-MH groups, receiver operating characteristic (ROC) curves were created using the R package pROC (version 1.19.0.1). For each eligible biomarker, 2-parameter logistic regression was performed using the glm function in R (version 4.3.0), and predicted values obtained from the logistic regression coefficients (maximum likelihood estimators) were used to create the ROC curve. The area under the curve (AUC), provisional cutoff value, sensitivity, and specificity were calculated from the ROC curve using the Youden method. Unless otherwise specified, statistical analyses were conducted using SAS 9.4 (SAS Institute Inc., Cary, NC).

## Results

### Demographic and clinical characteristics

Overall, 21 patients were included in the study, the median age at the time of informed consent was 46.0 years, 66.7% of patients were male, and the median body mass index was 20.90 kg/m^2^ (Table 1). Demographic and clinical characteristics for Week 14 CR (CR, *n* = 11; non-CR, *n* = 10) and MH groups (MH, *n* = 14; non-MH, *n* = 7) were similar. In more detail, 11 patients achieved both CR and MH, 3 patients achieved MH but did not achieve CR, and 7 patients did not achieve either CR or MH at Week 14 (Figure S1). Overall, 81.0% of patients had total colitis, and 81.0% of patients were bio-naïve (72.7% CR vs 90.0% non-CR; 78.6% MH vs 85.7% non-MH). All patients except for one had a history of steroid therapy, with 33.3% being steroid-resistant and 52.4% being steroid-dependent. Other characteristics were similar between patients who did and did not achieve CR or MH at Week 14. Patient characteristics were similar when stratified by CR and MH at Week 54 (Table S1).

**Table 1:** Demographic and clinical characteristics at baseline for all patients, and when stratified by CR and MH at Week 14.

Variable	All patients (N = 21)	CR assessment at week 14**^a^**		MH assessment at week 14**^a^**	
		CR (N = 11)	Non-CR (N = 10)	MH (N = 14)	Non-MH (N = 7)
**Age, median (Q1, Q3), years^b^**	46.0 (25.0, 60.0)	53.0 (25.0, 69.0)	46.0 (25.0, 51.0)	49.5 (25.0, 68.0)	46.0 (20.0, 51.0)
**Sex**					
** Male**	14 (66.7)	8 (72.7)	6 (60.0)	11 (78.6)	3 (42.9)
** Female**	7 (33.3)	3 (27.3)	4 (40.0)	3 (21.4)	4 (57.1)
**BMI, median (Q1, Q3), kg/m^2^**	20.90 (19.60, 25.70)	21.10 (19.60, 27.80)	19.85 (19.30, 24.70)	21.00 (19.60, 27.70)	19.80 (18.10, 24.70)
**Duration of disease, years**					
** ≥Min to <1**	1 (4.8)	0 (0.0)	1 (10.0)	0 (0.0)	1 (14.3)
** ≥1 to <3**	5 (23.8)	3 (27.3)	2 (20.0)	3 (21.4)	2 (28.6)
** ≥3 to <7**	3 (14.3)	2 (18.2)	1 (10.0)	3 (21.4)	0 (0.0)
** ≥7 to ≤ Max**	3 (14.3)	1 (9.1)	2 (20.0)	2 (14.3)	1 (14.3)
** Unknown**	9 (42.9)	5 (45.5)	4 (40.0)	6 (42.9)	3 (42.9)
**Disease classification by extent of UC lesions**					
** Total colitis**	17 (81.0)	8 (72.7)	9 (90.0)	11 (78.6)	6 (85.7)
** Left-sided colitis**	4 (19.0)	3 (27.3)	1 (10.0)	3 (21.4)	1 (14.3)
** Proctitis**	0 (0.0)	0 (0.0)	0 (0.0)	0 (0.0)	0 (0.0)
**History of treatment failure**					
** Biologics**					
** Bio-naïve**	17 (81.0)	8 (72.7)	9 (90.0)	11 (78.6)	6 (85.7)
** Corticosteroids**					
** Resistance**	7 (33.3)	3 (27.3)	4 (40.0)	5 (35.7)	2 (28.6)
** Dependence**	11 (52.4)	8 (72.7)	3 (30.0)	9 (64.3)	2 (28.6)
** Intolerance**	3 (14.3)	0 (0.0)	3 (30.0)	0 (0.0)	3 (42.9)
** No history**	1 (4.8)	1 (9.1)	0 (0.0)	1 (7.1)	0 (0.0)
** Immunomodulators**					
** No history**	14 (66.7)	6 (54.5)	8 (80.0)	9 (64.3)	5 (71.4)
** Tofacitinib**					
** No history**	20 (95.2)	11 (100.0)	9 (90.0)	14 (100.0)	6 (85.7)
**Partial Mayo score, mean (SD)**	5.4 (1.0)	5.2 (1.0)	5.7 (1.1)	5.2 (1.0)	5.9 (1.1)
**Full Mayo score, mean (SD)**	7.4 (1.3)	7.0 (1.0)	7.8 (1.6)	7.1 (1.1)	8.0 (1.6)
**Laboratory data**					
** Hb, median, g/dL (Q1, Q3)**	13.3 (11.9, 14.1)	13.4 (13.1, 14.2)	12.9 (11.8, 13.8)	13.5 (13.1, 14.2)	12.8 (11.8, 13.6)
** Alb, median, g/dL (Q1, Q3)**	4.0 (3.7, 4.4)	4.0 (3.7, 4.4)	4.0 (3.6, 4.4)	4.0 (3.7, 4.4)	3.9 (3.5, 4.5)

Data are *n* (%) unless stated otherwise.

Abbreviations: Alb, albumin; BMI, body mass index; CR, clinical remission; Hb, hemoglobin; Max, maximum; MH, mucosal healing; Min, minimum; Q1/3, quartile 1/3; UC, ulcerative colitis.

a At Week 14, 11 patients achieved both CR and MH, 3 patients achieved MH but did not achieve CR, and 7 patients did not achieve either CR or MH.

b At the time informed consent was obtained.

### Time course of partial Mayo scores

**Figure 1:**
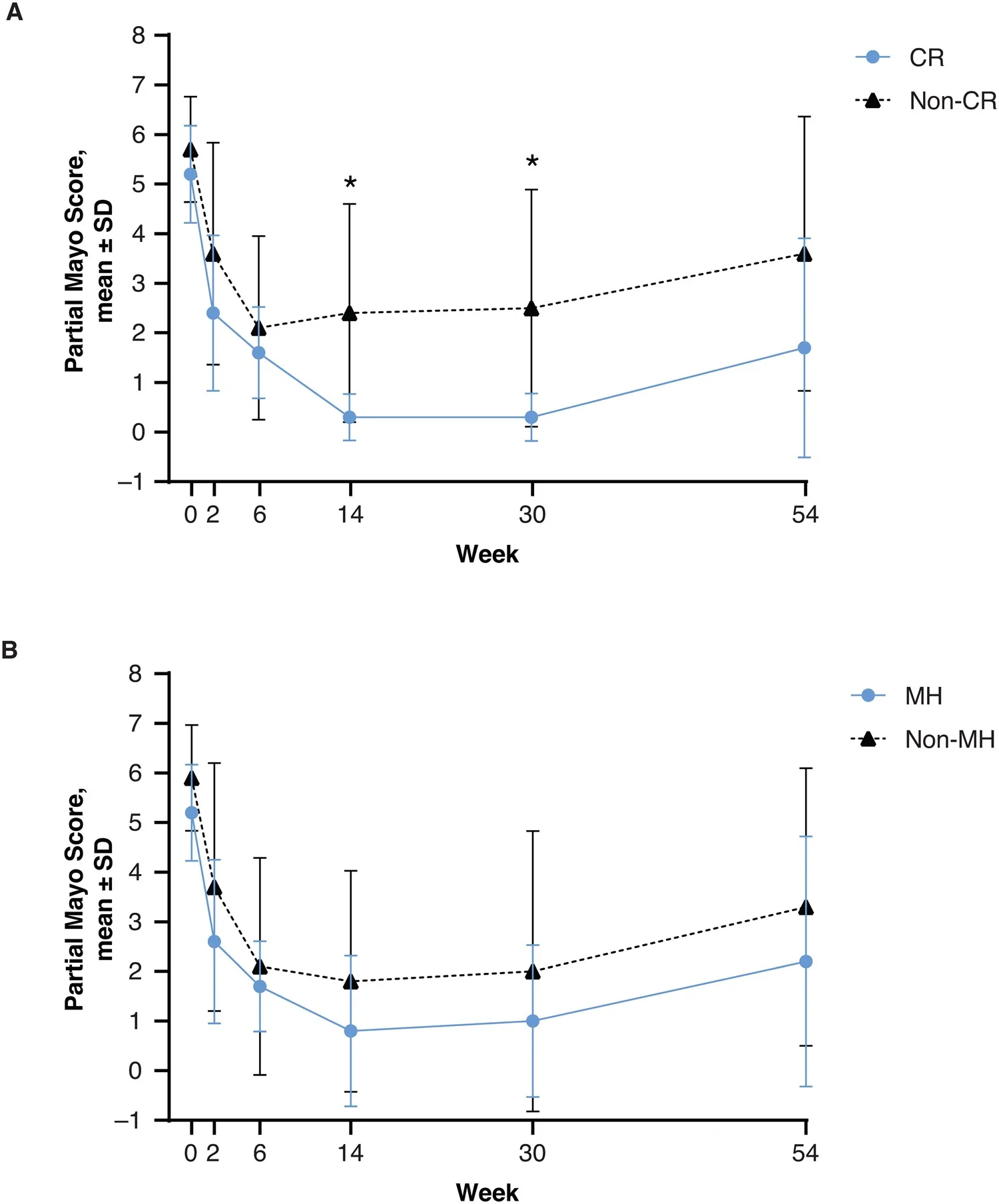
Time course of partial Mayo score in patients who did and did not achieve (A) CR or (B) MH at Week 14. *P < .05. CR, clinical remission; MH, mucosal healing.

The study compared partial Mayo score progression between the subgroups of patients who did and did not achieve CR or MH at Week 14 (Figure 1) and at Week 54 (Figure S2). Partial Mayo scores were significantly different between the CR and non-CR groups at Week 14 (mean [SD] partial Mayo score: 0.3 [0.47] vs 2.4 [2.20]; CR *n* = 11, non-CR *n* = 8; *P* = .0307) and at Week 30 (0.3 [0.48] vs 2.5 [2.39]; CR *n* = 10, non-CR *n* = 8; *P* = .0355); there were no significant differences in partial Mayo scores between the Week 14 MH and non-MH groups (Figure 1). Partial Mayo scores were significantly different at Week 54 between the CR and non-CR groups (0.6 [0.88] vs 4.6 [2.13]; CR *n* = 9, non-CR *n* = 9; *P* < .001) and between the MH and non-MH groups (1.4 [2.01] vs 4.4 [2.37]; MH *n* = 11, non-MH *n* = 7; *P* = .0159) (Figure S2).

### Serum biomarkers and FC levels

#### Comparison by CR and MH classifications at Week 14

**Figure 2:**
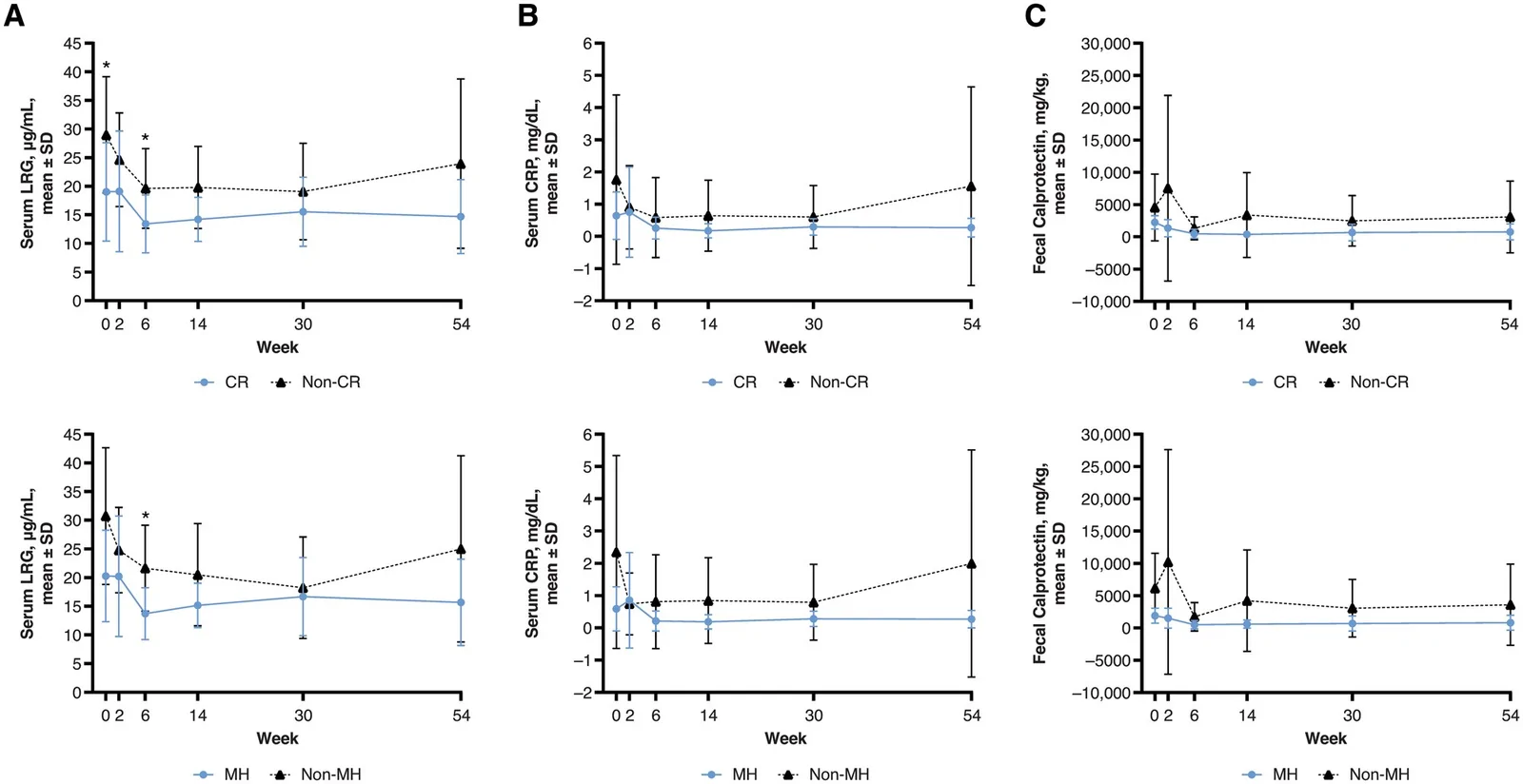
Time course of biomarkers (A) LRG, (B) CRP, and (C) fecal calprotectin by CR and MH at Week 14. *P < .05. CR, clinical remission; CRP, C-reactive protein; LRG, leucine-rich α-2-glycoprotein; MH, mucosal healing.

Biomarker profiles for the subgroups of patients who did and did not achieve CR or MH at Week 14 are shown in Figure 2 and Figure S3. There were significant differences in mean serum LRG levels between the CR and non-CR groups at Week 0 (19.0 vs 29.0 µg/mL; CR *n* = 11, non-CR *n* = 10; *P* = .0267) and Week 6 (13.4 vs 19.6 µg/mL; CR *n* = 11, non-CR *n* = 10; *P* = .0340), and between the MH and non-MH groups at Week 6 (13.7 vs 21.6 µg/mL; MH *n* = 14, non-MH *n* = 7; *P *= .0326) (Figure 2). The provisional cutoff value for serum LRG as a predictive biomarker (Week 0, stratified by CR) was 18.85 μg/mL (sensitivity = 63.6%, specificity = 100.0%, AUC = 0.827; Figure S4A). At Week 6, the provisional cutoff value stratified by CR was 13.2 μg/mL (sensitivity = 72.7%, specificity = 90.0%, AUC = 0.782; Figure S4B) and the provisional cutoff value stratified by MH was 17.5 μg/mL (sensitivity = 92.9%, specificity = 71.4%, AUC = 0.786; Figure S4C). There were no significant differences in serum CRP and FC levels at any time point between the CR or MH groups (Figure 2).

Serum MAdCAM-1 levels reached the lower limit (10.2 ng/mL) by Week 6 in all patients (Figure S3). A significant difference in serum MAdCAM-1 levels was observed between the MH and non-MH groups at Week 2 (11.7 vs 10.2 ng/mL; MH *n* = 14, non-MH *n* = 6; *P* = .0180). No significant differences in serum VDZ concentration were observed at any time point between the CR and non-CR or MH and non-MH groups (Figure S3).

**Figure 3:**
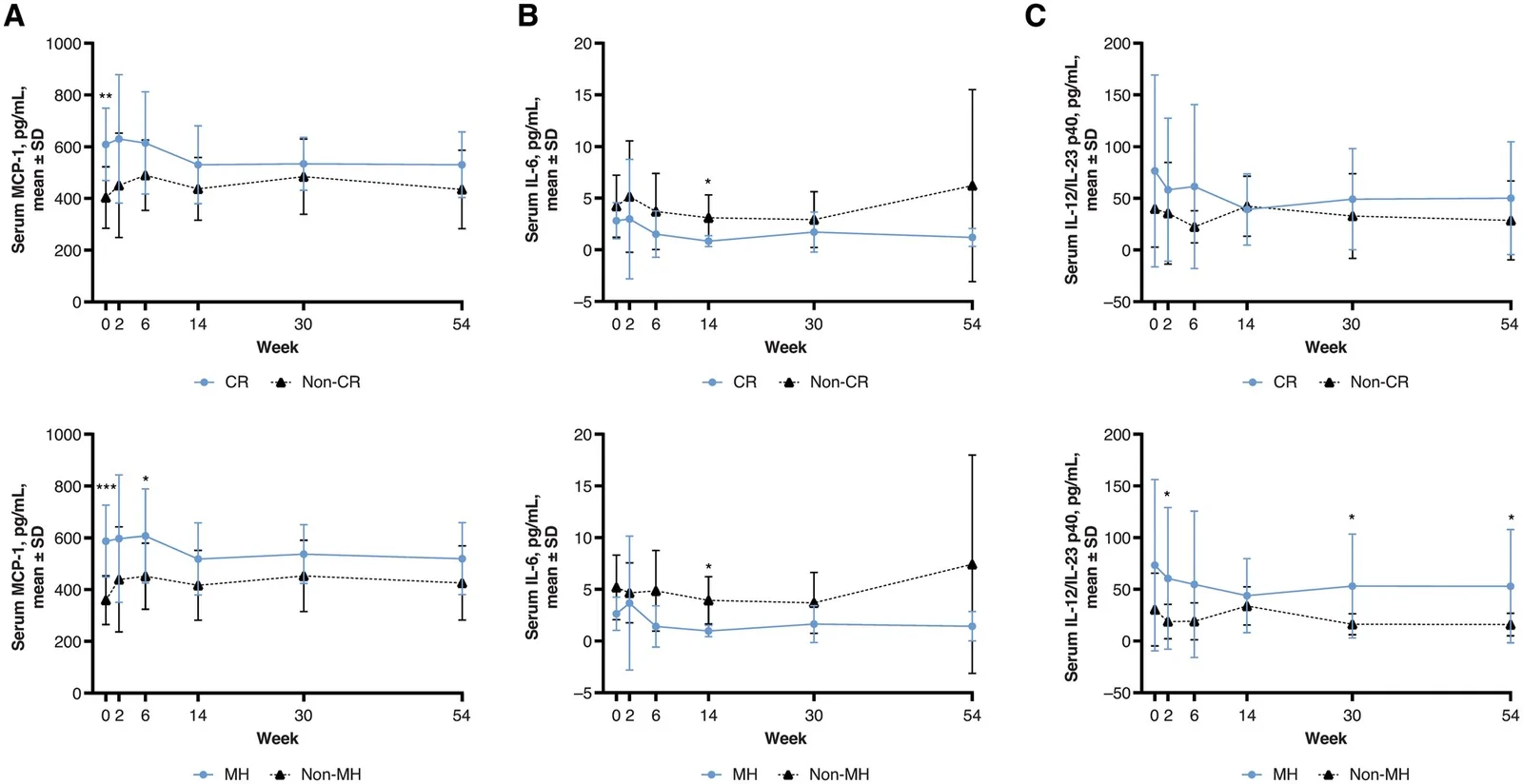
Time course of serum cytokines and chemokines (A) MCP-1, (B) IL-6, and (C) IL-12/IL-23 p40 by CR and MH at Week 14. *P < .05; **P < .005; ***P < .001. CR, clinical remission; IL, interleukin; MCP-1, monocyte chemoattractant protein-1; MH, mucosal healing.

Serum cytokine and chemokine profiles for the subgroups of patients who did and did not achieve CR or MH at Week 14 are shown in Figure 3 (MCP-1, IL-6, IL-12/IL-23 p40), Figure S5 (IL-1β, IL-8, IL-17A), Figure S6 (IFN-γ, TNF-α, OSM), and Figure S7 (IL-10, eotaxin, IL-1α, IL-2, IL-5, IL-13, IL-12 p70, IL-22, IL-17F, IL23 p19). There were significant differences in serum MCP-1 levels between the CR and non-CR groups at Week 0 (609.0 vs 403.4 pg/mL; CR *n* = 11, non-CR *n* = 10; *P* = .0018) and between the MH and non-MH groups at Week 0 (587.2 vs 358.9 pg/mL; MH *n* = 14, non-MH *n* = 7; *P* < .001) and Week 6 (606.9 vs 451.6 pg/mL; MH *n* = 14, non-MH *n* = 7; *P* = .037) (Figure 3). The provisional cutoff value for serum MCP-1 as a predictive biomarker (Week 0) was 467.75 pg/mL (stratified by CR: sensitivity = 90.9%, specificity = 80.0%, AUC = 0.864; Figure S8A; stratified by MH: sensitivity = 85.7%, specificity = 100.0%, AUC = 0.918; Figure S8B). At Week 6, the serum MCP-1 provisional cutoff value (stratified by MH) was 514.75 pg/mL (sensitivity = 64.3%, specificity = 85.7%, AUC = 0.745; Figure S8C). Significant differences in IL-6 levels were noted at Week 14 between the CR and non-CR groups (0.83 vs 3.10 pg/mL; CR *n* = 10, non-CR *n* = 9; *P* = .0151) and the MH and non-MH groups (0.97 vs 3.93 pg/mL; MH *n* = 13, non-MH *n* = 6; *P* = .0243) (Figure 3). Significant differences in IL-12/IL-23 p40 levels were noted between the MH and non-MH groups at multiple time points: Week 2 (60.5 vs 18.8 pg/mL; MH *n* = 14, non-MH *n* = 6; *P* = .0482), Week 30 (53.1 vs 16.1 pg/mL; MH *n* = 13, non-MH *n* = 6; *P* = .0235), and Week 54 (52.9 vs 15.9 pg/mL; MH *n* = 12, non-MH *n* = 6; *P* = .0431). For other cytokines, there were no significant differences at any time point between the CR or MH groups (Figures S5-S7).

#### Comparison by CR and MH classifications at Week 54

Biomarker profiles for the subgroups of patients who did and did not achieve CR or MH at Week 54 are shown in Figure S9. Significant differences in serum LRG were observed between the CR and non-CR groups at Weeks 0, 6, 30, and 54 (CR *n* = 9; non-CR *n* = 12 [Weeks 0, 6] and *n* = 9 [Weeks 30, 54]) and between the MH and non-MH groups at Week 30 (MH *n* = 11, non-MH *n* = 7). A significant difference in FC levels was observed between the CR and non-CR groups at Week 30 (CR *n* = 9, non-CR *n* = 8), and no significant differences in serum CRP were observed at any time point between the CR or MH groups. Serum MAdCAM-1 levels reached the lower limit (10.20 ng/mL) by Week 6 in all patients, and no significant difference in MAdCAM-1 levels was observed. Significant differences in serum VDZ concentration were observed between the CR and non-CR groups and the MH and non-MH groups at Week 30 (CR *n* = 9, non-CR *n* = 10; MH *n* = 11, non-MH *n* = 8) (Figure S9).

### Transcriptome analysis

#### DEGs in relation to CR and MH classifications at Week 14

**Figure 4:**
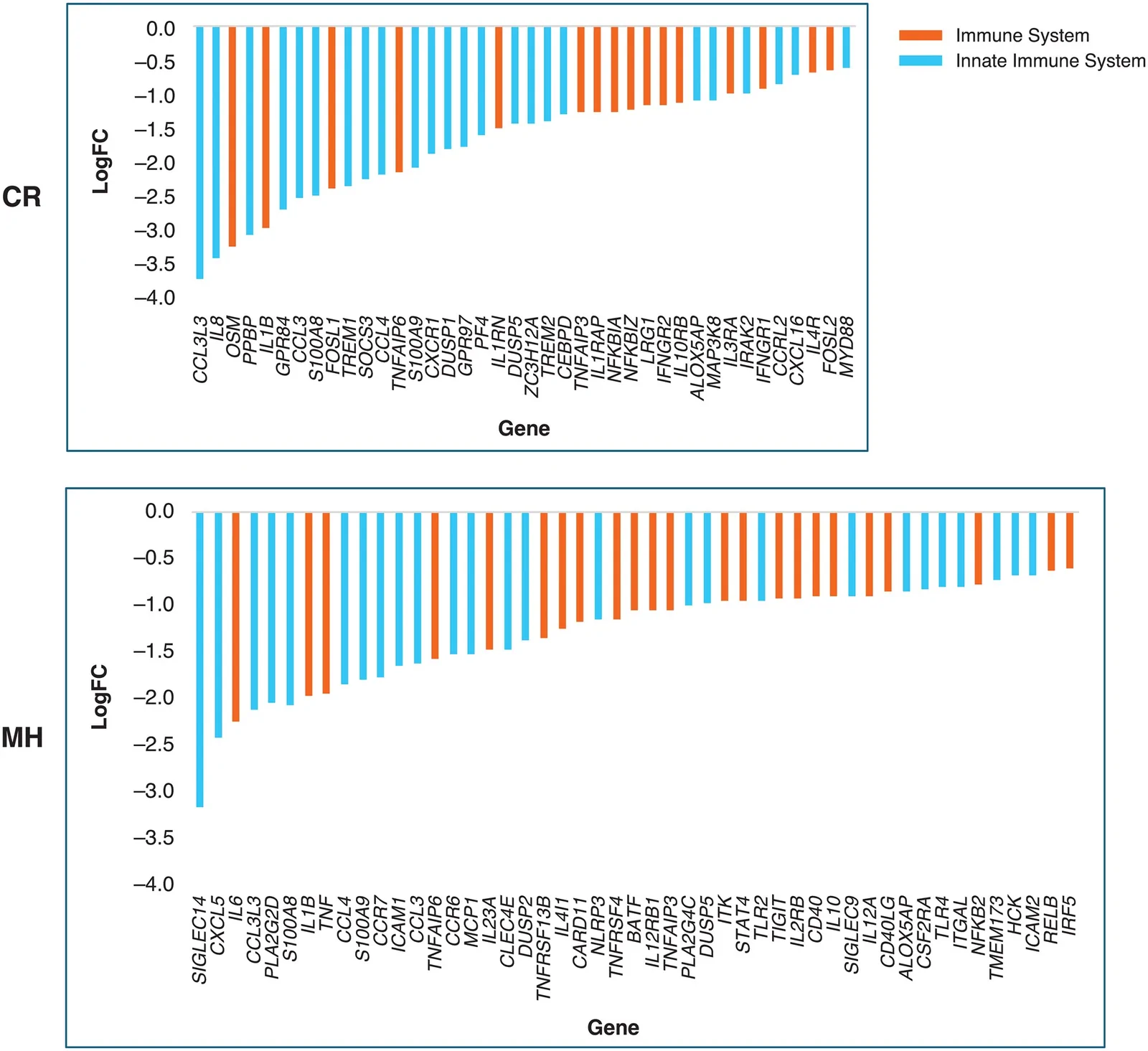
Log-fold changes in DEGs by CR and MH at Week 14. Innate immunity-related genes are shown in blue; immune-related genes are shown in orange. CR, clinical remission; DEG, differentially expressed gene; LogFC, log-fold change; MH, mucosal healing.

DEG extraction and enrichment analyses were performed on Week 0 inflammatory tissue and related to the VDZ treatment response (CR and complete-MH) at Week 14. The DEG analysis group included only 14 patients, as 7 patients had discontinued the study by Week 14 or tissue samples could not be collected. In 1 patient, CR at Week 14 could not be determined as only the Mayo endoscopic subscore was available and other subscores were unknown; this patient was excluded from the CR versus non-CR analysis. For the CR group (*n* = 10) and the non-CR group (*n* = 3), DEG extraction revealed an increased expression of 483 genes and a decreased expression of 487 genes in the CR group compared with the non-CR group. For the complete-MH group (*n* = 8) and non-complete-MH group (*n* = 6), DEG extraction revealed an increased expression of 148 genes and a decreased expression of 405 genes in the complete-MH group compared with the non-complete-MH group. Of all extracted DEGs, genes related to innate immunity and the immune system (identified through reviewing publicly available resources such as the PubMed database, all of which were downregulated genes) were selected, and their log-fold changes (logFC) are shown in Figure 4. Inflammatory cytokines (*IL6*, *OSM*, *IL1B*, *TNF*, *IFNGR*, *NFKBIA*, *IL23A,* etc.) and *LRG1* were identified. In addition, genes related to macrophage activation, chemokines and innate immunity (*SIGLEC14*, *IL8*, *CCL3*, *CCR6*, *SOCS3*, *S100A9,* etc.), and *MCP1* were identified (Table S2). DEGs common to both CR and complete-MH included *IL1B*, *CCL3*, *CCL3L3*, *S100A9*, *CCL4*, *TNFAIP6*, *DUSP5*, *TNFAIP3*, and *ALOX5AP*.

Enrichment analysis of these DEGs, using the Reactome_2022 library, revealed several common terms for the DEGs downregulated in the remission groups compared with the non-remission groups. The common terms between CR and complete-MH included immune system, innate immune system, neutrophil degranulation, IL-10 signaling, and cytokine signaling in the immune system (Figure S10).

**Figure 5:**
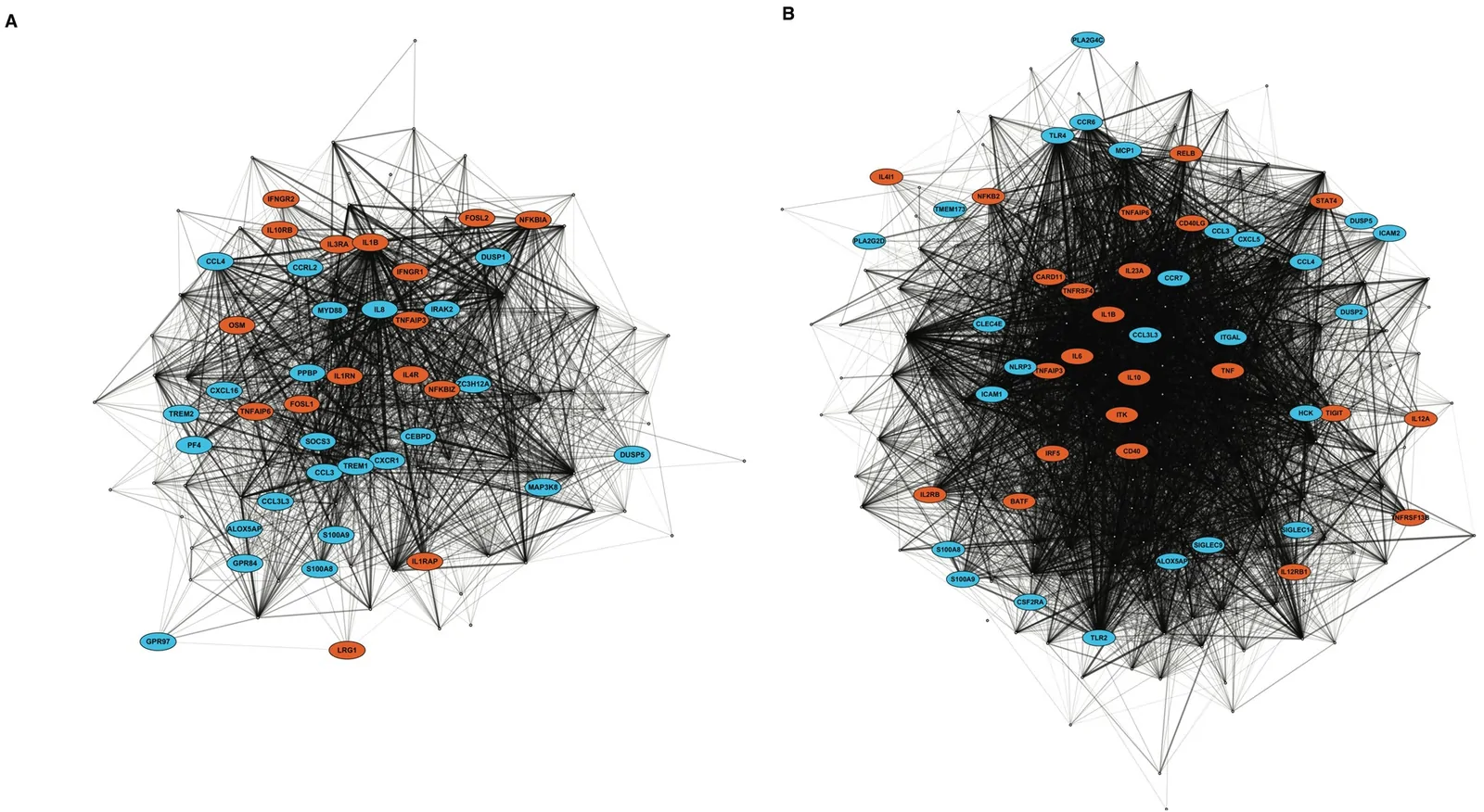
Network analysis: clusters of DEGs by (A) CR and (B) MH at Week 14. Innate immunity-related genes are shown in blue; immune-related genes are shown in orange. CR, clinical remission; DEG, differentially expressed gene; MH, mucosal healing.

Network analysis of DEGs was conducted using STRING v11.5 and Cytoscape 3.10.1 software to identify clusters containing *LRG* and *MCP1* in the CR and the complete-MH groups. Only innate immunity (blue) and immunity (orange) were then indicated (Figure 5).

#### Categorization of extracted DEGs

STRING-based network inference of the downregulated DEGs revealed innate immune system genes and immune system genes (Table 2). Innate immune system genes included those related to inflammation-induced macrophages, chemokines, chemokine receptors, innate cytokines and receptors, regulators of innate effector functions, and cell adhesion genes. Immune system genes included those related to Th1 signature, Th2 signature, Th17 signature, other inflammatory genes, and regulation of immunity. In addition, VDZ-specific integrin-related genes (*ICAM1*, *ICAM2*, *ITGAL*) were extracted.

**Table 2:** Categorizations of extracted DEGs.

**Innate immune system**										
** Macrophage activation**	*SIGLEC14*	*SIGLEC9*	*CEBPD*	*CLEC4E*	*ZC3H12A*	*TREM1*	*TREM2*	*CSF2RA*		
** Chemokines**	*CCL3L3*	*CCL3*	*CCL4*	*MCP1*	*CXCL16*	*PPBP*	*CXCL5*	*IL8*	*PF4*	
** Chemokine receptors**	*CXCR1*	*CCR6*	*CCR7*	*CCRL2*						
** Innate immune receptor**	*GPR84*	*GPR97*	*TLR2*	*TLR4*	*NLRP3*					
** Regulation of innate immunity**	*DUSP1*	*DUSP2*	*DUSP5*	*SOCS3*	*MAP3K8*	*IRAK2*	*TMEM173*	*MyD88*	*S100A9*	*S100A8*
** Cell adhesion**	*ICAM1,2*	*ITGAL*								
** Allergy**	*ALOX5AP*									
**Immune system**										
** Th1 signature**	*IFNGR1*	*IFNGR2*	*IL12RB1*	*NFKBIA*	*NRKBIZ*	*IL12RB1*	*IL2RB*	*STAT4*	*TNF*	
** Th2 signature**	*IL4I1*	*IL4R*	*IL10*	*IL10RB*						
** Th17 signature**	*IL23A*									
** Immune/inflammatory responses**	*IL1RN*	*IL3RA*	*IL6*	*IL1B*	*LRG1*	*OSM*	*FOSL1*	*FOSL2*		
** Regulation of immunity**	*IRF5*	*CARD11*	*ITK*	*RELB*	*TIGIT*	*BATF*				

Abbreviation: DEG, differentially expressed gene.

## Discussion

To implement personalized treatment strategies, it is necessary to identify biomarkers associated with treatment response to advanced therapies. In the present study, we investigated potential predictive biomarkers for VDZ treatment and found that baseline serum LRG levels differed significantly between patients who did and did not achieve CR at Week 14. In addition, among the serum cytokines and chemokines analyzed, MCP-1 was identified as a candidate biomarker associated with remission induction following VDZ treatment. Exploratory transcriptomic analyses suggested biological differences between patients who achieved CR and those who did not. Overall, our findings raise the possibility that baseline serum LRG and MCP-1 may be associated with response to VDZ treatment and warrant further investigation in larger, independent cohorts.

LRG is a glycoprotein expressed in localized inflammatory intestinal epithelial cells and is produced by neutrophils, macrophages, hepatocytes, and intestinal epithelial cells in response to IL-1β, IL-6, TNF-α, and IL-22. Unlike CRP, which is mediated solely by IL-6, serum LRG involves an IL-6-independent pathway.^11^ Serum LRG levels significantly correlate with clinical and endoscopic disease activities in patients with UC.^12^^,^^13^ Shinzaki et al found that in patients with IBD treated with adalimumab, endoscopic activity correlated better with serum LRG than with CRP or FC.^8^ Amano et al reported that serum LRG at Week 0 can predict the effectiveness of ustekinumab treatment at Week 8 in patients with IBD.^7^ Our study results suggest that serum LRG values reflect the therapeutic effect of VDZ more acutely than CRP or FC in patients with UC. Serum LRG levels appear to be effective biomarkers for predicting MH and CR at 14 weeks (Figure 2), as well as long-term CR at 54 weeks (Figure S9). Importantly, pretreatment serum LRG values may allow for sensitive prediction of CR versus non-CR, and serum LRG values at Week 6 may also serve as a useful biomarker reflecting the effectiveness of VDZ treatment.

This study also suggests that pretreatment serum levels of MCP-1 may potentially serve as a predictive biomarker for the therapeutic response to VDZ. Higher baseline levels of MCP-1 were observed in patients with CR and MH at Week 14 (Figure 3), with a provisional cutoff value of 467.75 pg/mL, which requires verification. To our knowledge, MCP-1 has not previously been suggested as a possible predictive biomarker for VDZ treatment response. MCP-1, in conjunction with its receptor CCR2, acts as a potent chemotactic and activating factor for monocytes and macrophages, playing a critical role in colitis by recruiting and activating these immune cells.^14^^,^^15^ Recent findings suggest that approximately 5% of non-classical monocytes express α4β7 integrin, which mediates dynamic adhesion to MAdCAM-1 and facilitates homing to the gut.^16^ These observations raise the possibility that MCP-1-mediated recruitment of monocyte populations capable of α4β7-dependent trafficking may influence therapeutic responses to VDZ.^17^ Although biomarkers related to the α4β7–MAdCAM-1 axis, such as serum MAdCAM-1 levels and serum VDZ concentrations, have been examined,^18^ consistent results regarding their predictive value have not been obtained.^19–22^ In this study as well, pretreatment MAdCAM-1 levels and VDZ concentrations were not effective in predicting treatment response. Further studies are needed to clarify the mechanistic relationship between MCP-1-associated immune cell trafficking and response to VDZ therapy.

In the gene expression analysis, regulators of Th1, Th2, and Th17 signatures, along with T-cell immunity, were associated with VDZ treatment, suggesting a possible direct or indirect action of the drug. LRG1-related genes reportedly include *OSM*, *IL22*, *IL6*, *TNFA*, *IL1B*, *LPS*, *IL17*, *IL4*, *IL33*, *IL10*, and *TGFB1*.^23^ Notably, in the current study, *OSM*, *IL1B*, *IL4*, *IL10*, *IL10R*, *NFKBIA*, and *FOSL1* were identified as DEGs, with *LRG1* itself included in the CR-based DEGs. These gene expression findings are consistent with the identification of baseline serum LRG as a candidate biomarker for VDZ treatment response.

Several cytokines, including IL-6, IL-12, and IL-23, are implicated in the pathogenesis of IBD,^24–26^ and IL-6 has been previously reported as a predictive biomarker in patients with UC treated with VDZ.^24^^,^^27^^,^^28^ In our study, serum levels of IL-6 or IL-12/IL-23 p40 at Week 0 were not associated with CR or MH, suggesting that these serum cytokines may not be reliable predictive biomarkers. However, there were significant differences in IL-6 and IL-12/IL-23 p40 between CR and non-CR groups and/or between MH and non-MH groups at later time points. Furthermore, the IL-6 gene (logFC –2.25, *P* = .015), *IL12A* (logFC –0.90, *P* = .039), and *IL23A* (logFC –1.49, *P* = .008) genes were among the downregulated DEGs in the complete-MH group (compared with the non-complete-MH group).

Other DEGs were identified in intestinal mucosa, despite there being no significant differences between the remission and non-remission groups in serum biomarkers. The genes *OSM*, *IL1B*, and *IL8* were all downregulated in intestinal tissue from patients with therapeutic response to VDZ. Specifically, *OSM* was downregulated in the Week 14 CR group (logFC –3.28, *P* = .0002), *IL1B* was downregulated in the Week 14 CR and complete-MH groups (logFC –3.01, *P* = .009 for CR, logFC –1.98, *P* = .029 for complete-MH), and *IL8* was downregulated in the Week 14 CR group (logFC –3.43, *P* = .027). However, the associated serum biomarkers, OSM, IL-1β, and IL-8, were not significantly different between patients with and without CR or MH at Week 14. These findings suggest that further investigation of OSM, IL-1β, and IL-8 may improve our understanding of their roles in both IBD activity and intestinal tissue response to VDZ therapy.

Recent studies have reported that VDZ significantly affects innate immunity, leading to changes in macrophage populations and microbial sensing.^29^ A previous analysis of VDZ-related genes from mucosal tissue highlighted dysregulated pathways in non-responders, including immune cell trafficking, the TNF receptor superfamily, the NF-kB pathway, interleukin signaling, MYD88 signaling, and toll-like receptors.^30^ Our study identified inflammation-induced macrophage-related genes (*SIGLEC14*, *CEBPD*, *CLEC4E*, *SIGLEC9*) and the inflammation enhancer *S100A8/9*, which collectively support the role of macrophage-related innate immunity in response to VDZ treatment. Among the DEGs identified in the MH group, *SIGLEC14* had the greatest logFC and has previously been shown to regulate macrophage immune responses following the activation by NLRP3 inflammasomes.^31^ We also observed in the MH group a decrease in cellular transport-related genes, including integrins (*ICAM1*, *ICAM2*, *ITGAL*) and various chemokine receptors, as well as *ITK*, which is essential for chemokine signaling and gut homing.^32^ Our results are consistent with the previous analysis associating VDZ response with gene regulation of immune cell trafficking and interleukin signaling. This supports VDZ’s role in T-cell mobilization to inflammatory tissues and is associated with a significant downregulation of innate immune signatures. In addition, the identification of a mucosal signature of the innate immune system involving chemokines raises the possibility that MCP-1 may be a predictive biomarker for VDZ treatment response.

The limitations of this study include the limited number of participants. Although we were able to calculate cutoff values for serum LRG and serum MCP-1 levels, the reliability of the ROC analysis used for these calculations is affected by small sample sizes. Therefore, the cutoff values reported in this study should be considered provisional. In addition, only 3 patients in the study had been previously treated with TNF-α antibodies, which is unusual for patients with UC and may limit the generalizability of the results. Another limitation was that the specific biopsy sites could not be identified, and gene expression may vary depending on the biopsy site. Furthermore, the transcriptome analysis included only 3 patients in the non-CR group, limiting the robustness and generalizability of these findings. Finally, FDR correction was not applied to the 48-plex cytokine/chemokine panel or the transcriptome data analyses; therefore, the possibility of false-positive findings arising from multiple comparisons should be considered when interpreting these results.

## Conclusion

High serum MCP-1 levels and low serum LRG levels at Week 0 may represent candidate biomarkers for predicting CR and MH with VDZ treatment in patients with UC. Exploratory mucosal transcriptome analysis stratified by CR and complete-MH suggested differences in immune-related pathways, including LRG1-associated T-cell immunity and macrophage-associated innate immunity, between treatment outcome groups. These findings support further investigation of serum LRG and MCP-1 as candidate biomarkers of VDZ treatment response in patients with UC.

## Supplementary Material

otag084_Supplementary_Data

## Data Availability

The data underlying this article will be shared on reasonable request from the corresponding author.
