# Supplementary material for: Serum leucine-rich α-2-glycoprotein and monocyte chemoattractant protein-1 as novel biomarkers for predicting vedolizumab response in patients with ulcerative colitis
Source: Crohns Colitis 360. 2026 Aug 11;8(3):otag084. doi: 10.1093/crocol/otag084 (PMC13495107; doi:10.1093/crocol/otag084)
Supplement: Supplementary file 1 [file otag084_supplementary_data.docx]

SUPPLEMENTARY DATA

**TITLE**

Serum Leucine-Rich α-2-Glycoprotein and Monocyte Chemoattractant Protein-1 as Novel Biomarkers for Predicting Vedolizumab Response in Patients With Ulcerative Colitis

**LIST OF TABLES AND FIGURES**

**Supplementary Table 1.** Demographics when stratified by CR and MH at week 54.

**Supplementary Table 2.** Network analysis of DEGs in the CR group, and in the MH group at week 14.

**Supplementary Figure 1.** Patient flow chart illustrating CR and MH assessment groups at weeks 14 and 54.

**Supplementary Figure 2.** Time course of partial Mayo score in patients who did and did not achieve (A) CR or (B) MH at week 54.

**Supplementary Figure 3.** Time course of (A) serum MAdCAM-1 and (B) serum VDZ concentration in patients who did and did not achieve CR or MH at week 14.

**Supplementary Figure 4.** ROC curves for serum LRG. (A) Week 0, stratified by CR at week 14. (B) Week 6, stratified by CR at week 14. (C) Week 6, stratified by MH at week 14.

**Supplementary Figure 5.** Time course of (A) IL-1β, (B) IL-8, and (C) IL-17A by CR and MH at week 14.

**Supplementary Figure 6.** Time course of (A) IFN-γ, (B) TNF-α, and (C) OSM by CR and MH at week 14.

**Supplementary Figure 7.** Time course of serum cytokines and chemokines by CR and MH at week 14.

**Supplementary Figure 8.** ROC curves for serum MCP-1. (A) Week 0, stratified by CR at week 14. (B) Week 0, stratified by MH at week 14. (C) Week 6, stratified by MH at week 14.

**Supplementary Figure 9.** Time course of biomarkers in patients who did and did not achieve CR or MH at week 54.

**Supplementary Figure 10.** Enrichment analysis of downregulated genes obtained by comparison of week 0 inflammatory tissue stratified by CR and MH at week 14.

**Supplementary Table 1.** Demographics when stratified by CR and MH at week 54.

| **Variable** | **CR assessment at  week 54** | | **MH assessment at  week 54** | |
| --- | --- | --- | --- | --- |
|  | **CR**  **(N=9)** | **Non-CR**  **(N=12)** | **MH**  **(N=11)** | **Non-MH**  **(N=10)** |
| Age, median (Q1, Q3), years | 46.0  (28.0, 53.0) | 48.0  (21.5, 65.0) | 51.0  (28.0, 60.0) | 45.5  (20.0, 62.0) |
| Sex |  |  |  |  |
| Male | 6 (66.7) | 8 (66.7) | 7 (63.6) | 7 (70.0) |
| Female | 3 (33.3) | 4 (33.3) | 4 (36.4) | 3 (30.0) |
| BMI, median (Q1, Q3), kg/m^2^ | 21.10 (19.30, 26.10) | 20.40 (19.60, 25.20) | 21.20  (19.30, 26.10) | 19.85  (19.60, 24.70) |
| Duration of disease, years |  |  |  |  |
| ≥Min to <1 | 0 (0.0) | 1 (8.3) | 0 (0.0) | 1 (10.0) |
| ≥1 to <3 | 1 (11.1) | 4 (33.3) | 1 (9.1) | 4 (40.0) |
| ≥3 to <7 | 2 (22.2) | 1 (8.3) | 2 (18.2) | 1 (10.0) |
| ≥7 to ≤Max | 1 (11.1) | 2 (16.7) | 1 (9.1) | 2 (20.0) |
| Unknown | 5 (55.6) | 4 (33.3) | 7 (63.6) | 2 (20.0) |
| Disease classification by extent  of UC lesions |  |  |  |  |
| Total colitis | 6 (66.7) | 11 (91.7) | 8 (72.7) | 9 (90.0) |
| Left-sided colitis | 3 (33.3) | 1 (8.3) | 3 (27.3) | 1 (10.0) |
| Proctitis | 0 (0.0) | 0 (0.0) | 0 (0.0) | 0 (0.0) |
| Right-sided or segmental colitis | 0 (0.0) | 0 (0.0) | 0 (0.0) | 0 (0.0) |
| History of treatment failure |  |  |  |  |
| Corticosteroids |  |  |  |  |
| Resistance | 5 (55.6) | 2 (16.7) | 6 (54.5) | 1 (10.0) |
| Dependence | 4 (44.4) | 7 (58.3) | 5 (45.5) | 6 (60.0) |
| Intolerance | 0 (0.0) | 3 (25.0) | 1 (9.1) | 2 (20.0) |
| No history | 0 (0.0) | 1 (8.3) | 0 (0.0) | 1 (10.0) |
| Immunomodulators |  |  |  |  |
| No history | 6 (66.7) | 8 (66.7) | 8 (72.7) | 6 (60.0) |
| Biologics |  |  |  |  |
| No history | 7 (77.8) | 10 (83.3) | 9 (81.8) | 8 (80.0) |
| Tofacitinib |  |  |  |  |
| No history | 9 (100.0) | 11 (91.7) | 11 (100.0) | 9 (90.0) |
| Partial Mayo score, mean (SD) | 5.2 (1.1) | 5.6 (1.0) | 5.4 (1.0) | 5.5 (1.1) |
| Full Mayo score, mean (SD) | 7.1 (1.6) | 7.6 (1.1) | 7.3 (1.5) | 7.5 (1.2) |
| Laboratory data |  |  |  |  |
| Hb, median (Q1, Q3), g/dL | 13.6  (12.9, 14.8) | 13.2  (11.9, 13.6) | 13.5  (11.9, 14.8) | 13.3  (11.8, 13.8) |
| Alb, median (Q1, Q3), g/dL | 4.2  (3.8, 4.4) | 3.9  (3.7, 4.2) | 4.2  (3.6, 4.4) | 4.0  (3.8, 4.4) |

Data are n (%) unless stated otherwise.

Abbreviations: Alb, albumin; BMI, body mass index; CR, clinical remission; Hb, hemoglobin; Max, maximum; MH, mucosal healing; Min, minimum; Q1/3, quartile 1/3; UC, ulcerative colitis.

**Supplementary Table 2.** Network analysis of DEGs in the CR group, and in the MH group at week 14.

| **Analysis group** | **DEGs** |
| --- | --- |
| Clinical remission | *AGER, ALOX5AP, AQP9, AREG, ATF3, B2M, BCL10, BCL2A1, BCL6, BHLHE40, C2CD4A, C2CD4B, C8orf4, CAST, CCL3, CCL3L3, CCL4, CCRL2, CD58, CEACAM3, CEBPD, CLEC7A, CMKLR1, CSRNP1, CXCL16, CXCR1, DUSP1, DUSP12, DUSP5, DUSP6, EDN1, EMP1, EMR3, EREG, ERRFI1, ETS2, FCGR2A, FFAR2, FOSL1, FOSL2, FPR1, FPR2, G0S2, GADD45B, GPR84, GPR97, HBEGF, HCAR3, HLA-E, IER3, IER5, IFNGR1, IFNGR2, IL10RB, IL1B, IL1RAP, IL1RN, IL3RA, IL4R, IL8, IRAK2, IRS2, JUNB, KLF6, LIF, LILRA2, LRG1, MAP3K8, MCL1, MMP10, MYD88, NCF2, NFKBIA, NFKBIZ, NLRP3, NR4A2, NR4A3, NT5E, OSM, P2RX4, PCDHB9, PELI1, PELI2, PF4, PLAUR, PPARD, PPBP, PSORS1C1, PTGS2, RIPK1, S100A8, S100A9, SERPINB6, SLPI, SOCS3, SPP1, STC1, TAB2, TFF2, TNFAIP3, TNFAIP6, TNFRSF10D, TNFRSF11B, TNFRSF1A, TNFRSF21, TNFSF13, TNIP1, TRAF3IP2, TREM1, TREM2, TSC22D1, TSLP, VNN2, ZC3H12A, ZFP36* |
| Mucosal healing | *ABL2, ADAM19, ADORA2A, AIF1, AKR1C3, ALOX5AP, ANGPTL6, APBB1IP, ARHGAP15, ARHGAP25, ARHGDIB, BATF, BATF3, BCL2A1, BCL6, BLK, BTG2, BTK, BTLA, CARD11, ,, CCL3, CCL3L3, CCL4, CCR6, CCR7, CD19, CD1C, CD226, CD28, CD300C, CD3D, CD40, CD40LG, CD48, CD5, CD52, CD53, CD6, CD69, CD7, CD80, CD83, CD84, CD86, CFP, CLEC2D, CLEC4E, CLEC7A, CMA1, CMTM3, CR1, CR2, CRLF2, CSF2RA, CST7, CTSG, CTSH, CXCL5, CXCR5, CYFIP2, CYTIP, DOCK11, DOCK2, DOK3, DRAM1, DUSP2, DUSP5, EVI2A, FAIM3, FCER1G, FCHSD2, FCN1, FCRL2, FCRLA, FERMT3, FGF2, FLT3LG, FPR3, FRZB, G0S2, GADD45B, GMFG, GPR132, GPR174, GPR183, GPR84, HCAR3, HCK, HCST, HLA-DPB1, HSPA1B, ICAM1, ICAM2, IGSF6, IKZF3, IL10, IL12A, IL12RB1, IL1B, IL23A, IL2RB, IL4I1, IL6, INPP5D, IRF5, ITGA4, ITGAL, ITK, KLF12, KLHL32, LAMP3, LAYN, LILRA5, LPXN, LST1, LTA, LTB, LY86, MAP3K8, MAPK11, MARCH1, MCP1, MNDA, NCF1, NCF4, NCKAP1L, NFATC1, NFKB2, NFKBIA, NINJ1, NLRP3, NR4A3, OSM, P2RX7, P2RY10, P2RY13, PARVB, PARVG, PGF, PIK3CG, PIK3R5, PIM1, PITRM1, PLA2G2D, PLA2G4C, PLEKHO1, POU2AF1, POU2F2, PREX1, PRKCQ, PSTPIP1, PTAFR, PTGES, PTGIR, PTGS2, RAC2, RASGRP1, RELB, RHOH, RNASE6, RUNX1, RUNX3, S100A8, S100A9, SASH3, SELE, SELL, SEPT1, SERPINB9, SI, SIGLEC14, SIGLEC9, SIT1, SLCO3A1, SNX20, SOD2, SPI1, STAT4, STX11, SUCNR1, SUSD3, TAGAP, TANK, TESPA1, TIAM2, TIGIT, TLR2, TLR4, TMC6, TMEM119, TMEM173, TNF, TNFAIP3, TNFAIP6, TNFAIP8, TNFAIP8L2, TNFRSF13B, TNFRSF4, TNFSF11, TRAF3IP3, TREML2, TYROBP, UCP2, USP38, VAV3, WDFY4, YAF2, YOD1, ZFP36, ZNF80* |

Abbreviations: CR, clinical remission; DEG, differentially expressed gene; MH, mucosal healing.

**Supplementary Figure 1.** Patient flow chart illustrating CR and MH assessment groups at weeks 14 and 54.

Abbreviations: AE, adverse event; CR, clinical remission; MH, mucosal healing.

^a^1 patient was classified as non-CR or non-MH due to an AE. This patient was excluded from the gene expression analysis cohort.

^b^3 patients, including 2 patients with AEs and 1 patient with lack of efficacy during the maintenance phase, were classified as non-CR or non-MH.

**Supplementary Figure 2.** Time course of partial Mayo score in patients who did and did not achieve (A) CR or (B) MH at week 54. **P*<.05; ****P*<.001.

Abbreviations: CR, clinical remission; MH, mucosal healing.

**Supplementary Figure 3.** Time course of (A) serum MAdCAM-1 and (B) serum VDZ concentration in patients who did and did not achieve CR or MH at week 14. **P*<.05. Abbreviations: CR, clinical remission; MAdCAM-1, mucosal addressin cell adhesion molecule-1; MH, mucosal healing; VDZ, vedolizumab.

**Supplementary Figure 4.** ROC curves for serum LRG. (A) Week 0, stratified by CR at week 14. (B) Week 6, stratified by CR at week 14.
(C) Week 6, stratified by MH at week 14.

Abbreviations: CR, clinical remission; LRG, leucine-rich α-2-glycoprotein; MH, mucosal healing; ROC, receiver operating characteristic.

**Supplementary Figure 5.** Time course of (A) IL-1β, (B) IL-8, and (C) IL-17A by CR and MH at week 14.

Abbreviations: CR, clinical remission; IL, interleukin; MH, mucosal healing.

**Supplementary Figure 6.** Time course of (A) IFN-γ, (B) TNF-α, and (C) OSM by CR and MH at week 14.

Abbreviations: CR, clinical remission; IFN, interferon; MH, mucosal healing; OSM, oncostatin M; TNF, tumor necrosis factor.

**Supplementary Figure 7.** Time course of serum cytokines and chemokines by CR and MH at week 14. (A) IL-10, (B) eotaxin, (C) IL-1α, (D) IL-2, (E) IL-5, (F) IL-13, (G) IL-12 p70, (H) IL-22, (I) IL-17F, and (J) IL-23 p19.

Abbreviations: CR, clinical remission; IL, interleukin; MH, mucosal healing.

**Supplementary Figure 8.** ROC curves for serum MCP-1. (A) Week 0, stratified by CR at week 14. (B) Week 0, stratified by MH at week 14.
(C) Week 6, stratified by MH at week 14.

Abbreviations: CR, clinical remission; MCP-1, monocyte chemoattractant protein-1; MH, mucosal healing; ROC, receiver operating characteristic.

**Supplementary Figure 9.** Time course of biomarkers in patients who did and did not achieve CR or MH at week 54. (A) LRG, (B) CRP,
(C) fecal calprotectin, (D) serum MAdCAM-1, and (E) serum VDZ concentration. **P*<.05, ****P*<.001.

Abbreviations: CR, clinical remission; CRP, C-reactive protein; LRG, leucine-rich α-2-glycoprotein; MAdCAM-1, mucosal addressin cell adhesion molecule-1; MH, mucosal healing; VDZ, vedolizumab.

**Supplementary Figure 10.** Enrichment analysis of downregulated genes obtained by comparison of week 0 inflammatory tissue stratified by CR and MH at week 14. Abbreviations: CD28, cluster of differentiation 28; CR, clinical remission; GPCR, G protein-coupled receptor; GTPase, guanosine triphosphatase; –log, negative log; MAL, MyD88 adaptor-like; MAP, mitogen-activated protein; MH, mucosal healing; MyD88, myeloid differentiation primary response gene 88; NADPH, nicotinamide adenine dinucleotide phosphate; NFkB, nuclear factor kappa B; RAC1, ras-related C3 botulinum toxin substrate 1; RHO, ras homologue; TIRAP, toll/interleukin-1 receptor domain-containing adaptor protein; TLR, toll-like receptor; TNF, tumor necrosis factor; TNFR2, tumor necrosis factor receptor 2; TNFSF, TNF receptor superfamily; TRAF6, TNF receptor-associated factor 6; Vav1, vav guanine nucleotide exchange factor 1.
